# The COV-ED Survey: exploring the impact of learning and teaching from home on parent/carers’ and teachers’ mental health and wellbeing during COVID-19 lockdown

**DOI:** 10.1186/s12889-022-13305-7

**Published:** 2022-05-04

**Authors:** C Connor, N De Valliere, J Warwick, S Stewart-Brown, A Thompson

**Affiliations:** Clinical Trials Unit, University of Warwick, Coventry, CV4 7AL England

**Keywords:** Parents/carers, Families, Teachers, COVID, Teaching, Learning, Lockdown, Mental health, Emotional wellbeing

## Abstract

**Background:**

Following the emergence of COVID-19 in the UK, on March 18^th^ 2020 the majority of schools in England closed and families and teachers were tasked with providing educational support for children and adolescents within the home environment. Little is known, however, regarding the impact of remote teaching and learning on the mental wellbeing of parents/carers and teaching staff.

**Methods:**

The Coronavirus Education (COV-ED) online survey explored the practicalities of learning and teaching from home for 329 parents/carers and 117 teachers of 11–15 year old adolescents in England, during June/July 2020, and the associated impact on their mental wellbeing. Participants were recruited through schools and via University of Warwick social media channels. Data was analysed using a series of Multiple Linear and Multivariate Regressions.

**Results:**

Despite coping well with the challenges of remote learning, a third of teachers reported below average mental wellbeing on the Warwick-Edinburgh Mental Wellbeing Scale. Multivariate regression revealed that wellbeing was associated with access to resources and confidence to teach from home. Almost half of parents/carers surveyed reported below average wellbeing. Multivariate regression revealed that poor wellbeing was more common in those who were also working from home and who lacked support for their own mental health. Concerns about their child’s mental health and lack of access to electronic devices and workspace were also significantly associated with the mental wellbeing of parents/carers.

**Conclusions:**

Whilst young people’s mental health and wellbeing has, and continues to be a national priority, the mental health and wellbeing of the families and teachers supporting them has not previously been explored. Our survey population was of predominantly white British heritage, female and living in the West Midlands UK, therefore, findings should be treated with caution. Findings provide a snapshot of factors that may be of significance to families and schools in supporting the mental wellbeing of those tasked with learning from home. They will help i) increase knowledge and awareness with regard to future support of families and teachers during similar crises; ii) enable the design and development of practical solutions in the delivery of remote teaching and learning; and, iii) help address the mental wellbeing needs of those tasked with supporting adolescents.

**Supplementary Information:**

The online version contains supplementary material available at 10.1186/s12889-022-13305-7.

## Introduction

In January 2020, following confirmation of its first two official cases of the new coronavirus SARS-CoV-2 (COVID-19), the UK government began to implement a series of measures to help reduce transmission of the COVID-19 virus. In March 2020 a national ‘lockdown’ was announced and the population were asked to to ‘stay at home’, avoid social gatherings and to work from home, wherever possible [1].

Increased vigilance over COVID-19 led to the subsequent closure of schools and nurseries in the UK, requiring parents/carers and teachers to support children and adolescents with teaching and learning from home [2, 3]. Between March and July 2020, over 575 million days of schooling were lost across England [4]. School closures will have been especially challenging for adolescents; a period of great physical and psychological change [5] when half of all lifetime mental disorders are believed to emerge [6]. Alongside this disruption, which may have included the postponement of important educational milestones, adolescents will have had little or no face-to-face contact with peers and limited interaction with teachers. This loss of social,emotional and educational support may have led to a disintegration of their usual coping mechanisms to deal with stress and anxiety [7, 8]. Research exploring the psychological impact of COVID on adolescents in Australia has revealed that COVID-related worries, difficulties with online learning and family conflict all affected young people’s mental wellbeing; most severely in those already experiencing mental health difficulties [9]. Indeed, a recent survey of over two-thousand adolescents with a pre-existing mental health issue found that 80% believed the pandemic had exacerbated their condition [10]. Understandably, protecting the mental wellbeing of young people and supporting their education from home during the pandemic has been a priority. However, the impact on the mental wellbeing of those *providing this support* has not been fully examined.

Mental wellbeing is the positive end on the continuum of mental health, with mental illness situated at the negative end of this continuum. Mental wellbeing refers to the emotional, psychological and social wellbeing of individuals in terms of perceived happiness, satisfaction and interest in life, and the balance between positive and negative affect [11]. The mental wellbeing of parents/carers and teachers was the key focus of the present study. The broader concepts of economic and social wellbeing are not applied at the individual level, and were, therefore, not included in the study.

### Parents and carers

#### Learning from home

A recent survey in Czechia has suggested that, despite the many and conflicting demands on their time, parents and carers were managing well with the practicalities of supporting their child with learning from home during COVID-19 pandemic school closures. However, lack of time and technological issues associated with remote learning often hindered their efforts and were compounded with feelings of inadequacy with regard to their own teaching skills and knowledge [12]. The UK Government has produced guidance for parents/carers to support their child’s remote education during COVID-19 lockdown. This guidance provides usesful information for parents and carers in terms of curriculum needs, engaging a child or young person at home, supporting their mental wellbeing and gaining access to appropriate educational resources [2]. These guidelines are vitally important given that it is recommended that young people in key stages 3 and 4 (ages 11–16) receive 4–5 h of remote education per day.

#### Working from home

Pre-pandemic, around 5% of the UK workforce were believed to be working predominantly from home [13]; this is in stark contrast to the estimated 60% of the UK population now doing so [14]. This number will inevitably include parents/carers who, in addition to working from home, now have responsibility for providing 4–5 h of supported learning for their child [2]. The recent Co-SPACE study [15] has revealed that whilst parents/carers were particularly concerned about their children’s mental wellbeing and social isolation during the pandemic, the principle cause of their stress was work-related. For families, the radical change in *work-life* and added responsibility of supporting their child’s educational needs will have disrupted *home-life* and altered any previously set routines. Family relationships may have become particularly stressful [16] as families sought to meet the educational and emotional needs of adolescents and their own lockdown challenges, struggling to compartmentalise novel work schedules, navigate remote working tools and platforms and balance these with the needs of their child. Acombination of family and workplace stress has been shown to have a negative impact on parenting behaviours as well as mental wellbeing [17, 18].

Government guidelines have emphasised the need for parents/carers to look after their own mental wellbeing during the pandemic, in order to be able to provide the best support for their children [19]. The relationship between parental mental wellbeing and the mental wellbeing of children and young people is well established. The presence of psychological problems in parents/carers has been associated with more negative parenting behaviours, neglect and increased family dysfunction [20, 21]. Such difficulties can increase stress and increase the likelihood of greater incidence of poor mental health and behavioural problems in children and young people [22, 23].

The general advice offered to parents/carerswith regard to protecting their own mental wellbeing highlights the importance of routine, social support, engagment in physical activity, adequate sleep, healthy diet and early help-seeking if problems arise [19, 24–26]. Being able to uphold these during lockdown, however, may have been especially difficult for those home-working parents/carers whose self-care may not have been prioritised.

### Teachers

#### Teaching from home

Anecdotal evidence has emerged which offers a glimpse of the ways in which teachers worldwide have dealt with the practicalities of teaching from home, how they have been supporting their students to learn remotely and independently and ensure ‘*Schools Out, But Class’s On’* [27]. The swift transfer from classroom to home schooling in the UK since March 2020, and the ongoing uncertainty regarding the safety of returning to the classroom, has meant that teachers have had to become increasingly flexible in their teaching practice and heavily reliant on digital technology. Teachers needed to find new and innovative ways of working, using videoconferencing and online learning resources with little or no training with regard to remote learning [25]. In addition to the acquisition of these new skills, teachers were also faced with the challenge of maintaining the motivation and engagement of students and creating a safe space in which adolescents could continue to thrive. Mental wellbeing has become embedded within the ethos of the majority of schools in England, but due to the pandemic, teachers were now placed in the unique position of having to do this remotely. General guidance for teachers to help adolescents cope during ‘hard times’ emphasises the important role of safeguarding [26]. To help adolescents cope with the unsettled daily routines, uncertainty and anxiety, teachers are urged to imbue a sense of connectedness and calm in their students and promote feelings of safety and hope. This is an ongoing challenge in ‘normal’ circumstances but made even more demanding in a remote setting.

There is little advice and understanding, however, of how teachers themselves met these new and challenging expectations and, equally importantly, how they attended to their *own* mental wellbeing during the pandemic. Self-care is vital if teachers are to provide the highest quality educational and emotional support for the adolescents in their care. Findings from a survey of teachers highlighted the negative impact that work-related stress may have on their capacity to facilitate early identification and intervention strategies with regard to the mental wellbeing of their students [28]. A recent survey of teachers and young people in England and Wales concluded that poor mental wellbeing in teachers may impact on the mental wellbeing of their students [29]. Indeed our own teacher survey in 2017 found that poorer mental wellbeing in teachers may specifically compromise their capacity and willingness to provide student support for their mental wellbeing [28].

UNESCO [30] have emphasised the importance of access to strategies and coping mechanisms for teaching staff and warn that increased workload and unrealistic expectations may lead to greater numbers of teaching staff experiencing burnout. These concerns were echoed by the National Association of Schoolmasters Union of Women Teachers (NASUWT) who stressed that schools must prioritise the mental wellbeing of staff and attend to the pressures placed on them during the pandemic [25].

Given future pandemics are likely to emerge more frequently (IPBES report [31]) parents/carers and teachers will need to be adequately supported, equipped and trained for the delivery of remote learning. It is essential, therefore, that schools and policy makers have a clear understanding, not only of the practicalities and challenges of teaching and learning from home but also the impact it may have on the mental wellbeing of those tasked with doing so, in order to provide them with the appropriate support.

## Methods

### Design

An online survey of parents/carers and teachers of adolescents aged 11–15 years in England. The survey consisted of two sections; data of interest were:

- The practicalities of teaching and learning from home for parents/carers and teachers during COVID-19 lockdown.
- The mental wellbeing of parents/carers and teachers supporting learning from home during COVID-19 lockdown;

#### Study population

The study population of interest were parents/carers and teachers supporting the educational needs of adolescents aged 11–15 in England during lockdown in June – July 2020. The survey was restricted to England as Scotland, Wales and Northern Ireland were responsible for their own policies in relation to public health issues and had separate legislation regarding COVID-19 lockdown and school closures.

There were an estimated 9.2 million families with children living in England in 2019; families are defined as a married, civil partnered or cohabiting couple with children, or a lone parent/carer, with at least one child, who live at the same address [32]. At the time of the 2011 census; 83.5% of households with dependent children were of White heritage, 8.9% Asian, 4.8% Black, 1.6% Mixed heritage and 1.2% from Other ethnic groups [33]. There were 453,813 full-time teachers in England in the year 2019–20 [34]. A third of all teachers were aged between 30–39 and 14.3% of all teachers in England described themselves as being in a minority ethic group.

#### Recruitment

The survey was initially launched on June 8^th^ 2020 and promoted via Birmingham and Coventry school bulletins. This included 32 schools in Birmingham and Coventry, already known to the research team through previous school-based research. Schools were provided with a link to the survey and a participant information sheet to share with all families and teaching staff, either by email or preferred channels (e.g. school newsletter/intranet). Promotion was the responsibility of those schools who expressed interest in the survey and we had no capacity to monitor response rates from each site. On June 15^th^ 2020, a link to the survey was subsequently released online, via University of Warwick social media channels (Facebook, Twitter and Linked-in) allowing parents/carers and teachers across England to participate. Again, this methodology did not enable us to monitor response rates.

#### Inclusion criteria

The survey was conducted completely online, therefore, participants were required to have access to the internet, a computer/laptop/tablet/mobile phone and the capacity to understand the English language, or access to support, in order to complete the survey.

#### Exclusion criteria

There were no exclusion criteria. All participants, including those who had, or were currently suffering with COVID-19 were welcome to take part. However, we did not enquire about participants’ COVID-19 status in the survey.

#### Data collection

The survey was live for 54 days (June 8^th^ – July 31^st^ 2020). Consent, registration and survey data was collected online using the Qualtrics survey platform [35]. All data collected was anonymised and no personal data was collected.

The survey took approximately 15-min to complete and was accessible via computer, laptop, tablet or mobile phone to enable flexibility and accessibility. Once registered, participants had 7-days to complete their survey, and were able to save partially completed surveys and revisit them during the 7-day period. Any data which was incomplete after 7-days was automatically deleted and not included in the analyses.

#### Ethical approval

Ethical approval was granted by the University of Warwick Biomedical & Scientific Research Ethics Committee (BSREC 123/19–20).

### Measures

The survey consisted of two sections:

#### Part One – The practicalities of teaching/learning at home

The questions for Part One were specifically developed for the purposes of the online survey as no existing or validated measures which focused on the topics of interest were currently available. Practicalities of teaching and learning from home refer to the reality of the day-to-day experience of supporting young people in a remote setting.. To ensure content validity the survey (see Supplementary Tables 1 and 2, Additional File 1) went through several iterations which were reviewed by all members of the research team who are experienced in early intervention, youth, school and family emotional health research and design.

##### Teachers

Twenty-three survey questions explored teaching activities, numbers of students, hours of teaching, contact and engagement with students, families and school, access to resources and self-efficacy (see Supplementary Table 1, Additional File 1 for complete teacher survey).

##### Parents/carers

Twenty-two questions explored number and age of dependent children, number of hours per day spent supporting their child, home schooling day structure, access to resources (self and child), contact and engagement with child/children and their school, and self-efficacy (see Supplementary Table 2, Additional File 1 for complete parent/carer survey).

#### Part Two – Warwick-Edinburgh Mental Wellbeing Scale (WEMWBS [36])

The 14-item scale measures mental wellbeing in the general population. The items cover positive feeling and functioning aspects of mental wellbeing (e.g. *I’ve been feeling optimistic about the future; I’ve been feeling good about myself*) using a Likert scale (*none of the time; rarely; some of the time; often; all of the time*). All items are positively worded and total scores range between 14–70, with a higher score corresponding to a higher level of mental wellbeing. The scale extends the measurable range of mental wellbeing and correlates highly with other measures of mental wellbeing. It has been bench-marked on Centre for Epidemiologic Studies Depression Scale (CES-D [37]) with which it is highly correlated (0.84 [38]). The WEMWBS has a high level of internal consistency (α = 0.91) and test–retest reliability (0.83 (31)). A score of 41–44 is indicative of possible/mild depression; a score of < 41 is indicative of probable clinical depression [39], cut-points which can be used for analysis if focusing on likely mental illness. It is validated for use in a variety of settings. The minimally important difference calculated, using different statistical methods has been reported as between 3 and 8 points [40].

### Data management & protection

Data was collected via the online Qualtrics platform and was downloaded to a secure server at Warwick Clinical Trials Unit (WCTU). Following download, all data on Qualtrics platform was deleted. No personal data was collected or stored on the WCTU database or used for unrelated purposes. Data was only accessible to the research team.

### Statistical analysis

All analyses were undertaken using Stata 16 (StataCorp 2019).

Information on demographics, practicalities of teaching from home and supporting a child with learning from home were presented using basic descriptive statistics. Continuous data was summarised using mean and standard deviation (if normally distributed) and the median and interquartile range (if skewed). Categorical data was reported using the number and percentage of participants in each category. Multiple linear regressions were utilised to explore the effect of demographic variables and practical aspects of teaching from home on the mental wellbeing of teachers, and, similarly, to explore the effect of demographic variables and practical aspects of supporting a child with learning from home on the mental wellbeing of parents/carers. The dependent variable in the regression models was WEMWBS score with demographics and practicalities as independent variables. The normality of WEMWBS score was assessed graphically (through histogram plots). Diagnostic plots were also used to verify that the distributional assumptions of the regression model had been met. To reduce the number of variables to be included in the two regression models, two researchers (CC, NDV) identified the subset of questions that they thought would be most strongly related to mental wellbeing prior to seeing the data. Two questions in the teachers’ survey (Teacher Experience Q10 and Q11, Supplementary Table 1) regarding “*feeling supported by the management*” were thought to be very closely related to each other, so these were combined for inclusion in the regression models by adding together scores (full details are provided in Supplementary Table 3, Additional File 1). Similary, answers from the teachers’ survey on the two questions about “*feeling supported by colleagues*” (Teacher Experience Q12 and Q13, Supplementary Table 1) were combined (as described in Supplementary Table 3). Questions where respondents ‘*mostly agreed’* or ‘*strongly agreed’* were felt to be uninformative and unlikely to explain differences in outcome between participants and so were excluded from the regression models. Owing to small numbers, the last two categories of responses that were recorded using the five-level Likert scale (*strongly agree, agree, neutral, disagree, strongly disagree*) were combined (except for one in the parents survey, relating to the statement “*Working from home at the same time as supporting my child with learning is easy to coordinate”* where it was more appropriate to merge the first two categories). Variables considered for inclusion in the teachers regression model were gender, age category (< 45 years, ≥ 45 years), ethnicity (white, minority ethnicities), “*having someone to support you with your mental health*” (yes/no), “*feeling supported by management”* (high, moderate, low), “*feeling supported by colleagues*” (high, moderate, low), “*having adequate resources*”, “*having adequate training*”, “*having a desk or workspace*”, “*feeling confident and capable about teaching from home*”, “*having students that are engaged in online teaching*” and “*having students who are coping well with online teaching*”.

For the parents model, variables considered for inclusion in the regression model were sex, age category (< 45 years, ≥ 45 years), ethnicity (white, minority ethnicities), “*having someone else to support their child with learning from home”* (none, parent/grandparent/other family member), “*having someone to support them with their mental health*” (yes/no), “*having a structured home schooling day”,* “*having been provided with adequate home learning resources by their child’s school*”, “*using other resources for learning from home*”, “*child having access to their own laptop/computer/tablet/smart phone*”, “*working from home at the same time*”, “*finding working from home at the same time as suporting their child with learning easy to co-ordinate*”, “*having regular contact with their child’s school and/or teacher*”, “*feeling supported by their childs teacher/school*”, “*having their child’s school/teacher set regular deadlines*”, “*feeling capable and confident in supporting their child with learning*”, “*feeling capable and confident using technology to support their child’s learning*”,”*having a child that is engaged with and enjoying learning from home*””*belief that their relationship with their child has improved whilst learning from home*” and “*belief that their child’s mental health has been affected by learning from home*”. Univariable models were fitted and a parsimonious multivariable model built using forward selection. Robust cluster variance estimators were used for the parent/carer model, so that more than one child could be included per parent. Coefficients from the regression models are reported with associated p-values and 95% confidence intervals.

For both groups WEMWBS scores were grouped as follows [41], one standard deviation above the mean or more: above average (60–70); one standard deviation below the mean or more: below average (14–42); remainder: average (43–59). Below average scores indicate a high probability of clinical depression or anxiety.

## Results

### Participant demographics

A total of 501 participants completed the online survey. 138 teachers (Table 1) and 363 parent/carers (Table 2). 21 (15.2%) teachers were not teaching from home and 34 (10%) of parents/carers were not supporting a child with learning from home and were removed from the analysis. Our analysis, therefore, focused on 446 participants (117 teachers and 329 parent/carers). Demographic information is presented in Tables 1 and 2 respectively.

**Table 1:** Teachers’ demographics

	**Teachers who were not supporting a child with learning from home**	**Teacher who were supporting a child with learning from home**	**All teachers**
	***N* = 76**	***N* = 41**	***N* = 117**
**Gender**			
Female	60 (79.0%)	32 (78.1%)	92 (78.6%)
Male	16 (21.1%)	9 (22.0%)	25 (21.4%)
**Age**			
18–24	4 (5.3%)	0	4 (3.4%)
25–34	28 (36.8%)	2 (4.9%)	30 (25.6%)
35–44	14 (18.4%)	13 (31.7%)	27 (23.1%)
45–54	23 (30.3%)	24 (58.5%)	47 (40.2%)
55–64	6 (7.9%)	2 (4.9%)	8 (6.8%)
65 & over	1 (1.3%)	0	1 (0.9%)
**Ethnicity**			
White	65 (85.5%)	36 (87.8%)	101 (86.3%)
Minority ethnicities	9 (11.8%)	3 (7.3%)	12 (10.3%)
Unknown	2 (2.6%)	2 (4.9%)	4 (3.4%)
**School Region**			
West Midlands	68 (89.5%)	31 (75.6%)	99 (84.6%)
Other^a^	8 (10.5%)	8 (19.5%)	16 (13.7%)
Prefer not to answer	0	2 (4.9%)	2 (1.7%)
**Type of school**			
Academy	32 (42.1%)	15 (36.6%)	47 (40.2%)
Faith	17 (22.4%)	5 (12.2%)	22 (18.8%)
Grammar	5 (6.6%)	1 (2.4%)	6 (5.1%)
Independent/Private	6 (7.9%)	8 (19.5%)	14 (12.0%)
Special	7 (9.2%)	3 (7.3%)	10 (8.6%)
State/Maintained	5 (6.6%)	3 (7.3%)	8 (6.8%)
Other	4 (5.3%)	5 (12.2%)	9 (7.7%)
Prefer not to answer	0	1 (2.4%)	1 (0.9%)
**WEMWEBS score (mean, sd)**	46.41 (8.7)	46.02 (9.1)	46.27 (8.8)
**WEMWEBS score (categorical)**			
Above average (60–70)	5 (6.6%)	2 (4.9%)	7 (6.0%)
Average (43–59)	49 (64.5%)	26 (63.4%)	75 (64.1%)
Below average (17–42)	22 (28.9%)	13 (31.7%)	35 (29.9%)

a Other regions: East Midlands & East of England, London & South East, North England, South West

**Table 2:** Parents/carers’ demographics

	**Parents who were not teachers**	**Parents who were teachers**	**All parents**
	***N* = 284**	***N* = 45**	***N* = 329**
**Gender**			
Female	244 (85.9%)	37 (82.2%)	281 (85.4%)
Male	39 (13.7%)	8 (17.8%)	47 (14.3%)
Other/Unknown	1 (0.4%)	0	1 (0.3%)
**Age**			
18–24	0	0	0
25–34	9 (3.2%)	2 (4.4%)	11 (3.3%)
35–44	103 (36.3%)	16 (35.6%)	119 (36.2%)
45–54	153 (53.9%)	25 (55.6%)	178 (54.1%)
55–64	16 (5.6%)	2 (4.4%)	18 (5.5%)
65 & over	2 (0.7%)	0	2 (0.6%)
Unknown	1 (0.4%)	0	1 (0.3%)
**Ethnicity**			
White	257 (90.5%)	39 (86.7%)	296 (90.0%)
Minority ethnicities	25 (8.8%)	4 (8.9%)	29 (8.8%)
Unknown	2 (0.7%)	2 (4.4%)	4 (1.2%)
**Region**			
West Midlands	207 (72.9%)	33 (73.3%)	240 (72.9%)
Other^a^	75 (26.4%)	11 (24.4%)	86 (26.1%)
Unknown	2 (0.7%)	1 (2.2%)	3 (0.9%)
**WEMWEBS score (mean, sd)**	43.7 (9.6)	45.91 (8.2)	44.01 (9.44)
**WEMWEBS score (categorical)**^b^			
Above average (60–70)	16 (5.6%)	1 (2.2%)	17 (5.2%)
Average (43–59)	135 (47.5%)	30 (66.7%)	165 (50.1%)
Below average (14–42)	133 (46.8%)	14 (31.1%)	147 (44.7%)

^a^Other regions: East Midlands & East of England, London & South East, North England, South West

^b^Cut points are based on the UK population [32]

### Teachers

Teacher survey respondents were mostly female (78.6%), of white heritage (86.3%) and working in the West Midlands (84.6%). A large proportion (35%) were also supporting their own child (or children) with learning from home (teacher/parent/carer). Roughly one third of teachers (29.9%) reported mental wellbeing (WEMWEBS) scores that were below the average for the general UK population. This proportion did not differ substantially between those teachers who were also a parent/carer and those who were not.

### Parents/carers

Parent/carer participants were also predominantly female (85.4%), of white heritage (90%) and from the West Midlands (73%). A greater number (44.7%) reported below average mental wellbeing (as per WEMWBS score) compared with teachers. There was a marked diffference in mental wellbeing between the parents who were teachers and parents who were not, with 46.8% of parents who were not teachers reporting below average mental wellbeing compared to 31.1% of parents who were also teachers.

### The practicalities of learning/teaching from home

#### Teachers

Responses to the survey (*n* = 117) regarding the practicialities of teaching from home are summarised in Supplementary Table 4, Additional File 1. Most were teaching mixed gender classes and were engaged in online teaching for five or more hours per day. Over half had daily contact with their students and 74% were in contact with the parents/carers of their students. The great majority (92%) were having regular contact with senior management and colleagues at their school with regard to home teaching. 69% having been provided with adequate resources to teach from home and 62% having received adequate training to do so. 79% felt confident and capable to teach from home and 86% said they had adapted well to the technology necessary to do so. 52.1% felt that their students were engaged in online teaching. All teachers reported having someone to support them with their mental wellbeing.

#### Parents/carers

Responses to the survey (*n* = 329) on the family level practicalities of supporting a child/children with learning from home are summarised in Supplementary Table 5a, Additional File 1. The majority of respondents (68.4%) were supporting only one child with learning from home and mostly (62%) with the help of a partner. 209 (63.5%) reported having someone to support them with their mental wellbeing g.

Responses to the survey on the child-level practicalities of supporting a child/children with learning from home are summarised in Supplementary Table 5b, Additional File 1. As the majority were supporting only one child, key data from the column headed “Child 1” are highlighted. 191 (58.1%) felt they had been given adequate resources from their child’s school. 235 (71.4%) were also having to work from home. 142 (43.5%) said they felt confident and capable in teaching from home. 129 (39.2%) reported that their child was engaged in learning from home. 204 (62%) reported that their child’s mental wellbeing had been affected by learning from home.

#### Mental wellbeing of teachers

Estimates from the multivariable linear regression model used to explore the effect of demographic variables and practical aspects of teaching from home on the mental wellbeing of teachers are presented in Table 3 (estimates from the univariable model are presented in Supplementary Table 6, Additional File 1).

**Table 3:** Multivariable linear regression model exploring effect of teacher survey responses on teachers’ mental wellbeing

	**Multivariable model**	
**Factor**	**Coefficient (95% CI)**	***p*-value**
**My school has provided me with adequate resources to teach from home**		
Strongly agree	Ref	
Agree	-2.10 (-5.97– 1.77)	
Neutral	-4.54 (-9.10 – 0.02)	
Disagree/Strongly disagree	-8.49 (-14.17 – -2.80)	0.02
**I feel confident and capable with regard to teaching from home**		
Strongly agree	Ref	
Agree	-1.46 (-5.15 – 2.23)	
Neutral	-0.95 (-6.04 – 4.14)	
Disagree/Strongly disagree	-9.71 (-16.54 – -2.87)	0.04

Only the questions “*My school has provided me with adequate resources to teach from home”* and “*I feel confident and capable with regard to teaching from home*” were significant in the multivariable models but the effect sizes were slightly reduced and *p*-values increased, suggesting that answers to the two questions were related to each other although both have independent effects. Thus, our best estimates were that teachers who disagreed with the former statement were, on average, 8.49 points lower on WEMWEBS (95% CI -14.17 – -2.80) (*p* = 0.02) than teachers who strongly agreed with the statement. Teachers who disagreed with the latter statement scored 9.71 points lower (95% CI -16.52 – -2.87) than teachers who agreed with the statement.

#### Mental wellbeing of parents/carers

Estimates from the multivariable linear regression model used to explore the effect of the demographic variables and survey responses on the mental wellbeing of parents are presented in Table 4 (estimates from the univariable model are presented in Supplementary Table 7, Additional File 1).

**Table 4:** Multivariable linear regression model exploring effect of parent survey responses on parents’ mental wellbeing

	**Multivariable model**	
**Factor**	**Coefficient (95% CI)**	***p*-value**
**I have someone to support me with my mental health and wellbeing**		
No	Ref	
Yes	4.15 (2.19 – 6.11)	< 0.001
**My child has access to their own laptop/computer/tablet/smartphone to complete school work**		
Strongly agree	Ref	
Agree	-0.22(-2.08 – 1.64)	
Neutral	-1.78 (-5.80 – 2.24)	
Disagree/strongly disagree	-5.87 (-8.85 – -2.88)	0.002
**Working from home at the same time as supporting my child with learning is easy to coordinate**		
Strongly agree/agree	Ref	
Neutral	-2.54 (-5.29 – 0.20)	
Disagree	-2.86 (-5.32 – -0.40)	
Strongly disagree	-6.28 (-8.85 – -3.61)	< 0.001
**I feel supported by my child’s school and/or teacher and know who to contact if I have any problems with learning from home**		
Strongly agree	Ref	
Agree	-4.49 (-7.16 – -1.82)	
Neutral	-4.29 (7.22 – 1.37)	
Disagree/strongly disagree	-5.46 (-8.34 – -2.57)	0.003
**I feel capable and confident using technology to support my child with learning from home**		
Strongly agree	Ref	
Agree	-2.32 (-4.65 – 0.00)	
Neutral	-2.31 (-5.12 – 0.50)	
Disagree/strongly disagree	-5.02 (-8.03 – -2.01)	0.01
**My child’s mental health and wellbeing has been affected by learning from home**		
No	Ref	
Yes	-4.25 (-6.11 – -2.38)	< 0.001

Having someone to support them with their mental wellbeing increased parents’ WEMWEBS scores by 4.15 points on average (95% CI 2.19 – 6.11) compared to those who did not. Those who disagreed with the statement “*My child has access to their own laptop/computer/tablet/smartphone to complete schoolwork”* scored 5.87 points lower (95%CI -8.95 – -2.88) than those who strongly agreed. Those who disagreed with the statements”*Working from home at the same time as supporting my child with learning is easy to coordinate”, “I feel supported by my child’s school and/or teacher and know who to contact if I have any problems with learning from home”, “I feel capable and confident in supporting my child with learning from home.”, “I feel capable and confident using technology to support my child with learning from home” and “My relationship with my child has improved whilst learning from home*” scored 6.28 points lower (95% CI -8.85 – -3.61) on average than those who strongly agreed and those who answered yes to “*My child’s mental health and wellbeing has been affected by learning from home*” are independent predictors of the mental wellbeing of parents who are supporting their child with learning at home (scored 4.25 points less per WEMWEBS on average than those who answered no).

## Discussion

### Practicalities of teaching from home for teachers

Overall, we found that teachers had coped well with remote teaching and had adapted to the novel and challenging situation, despite some having responsibility for supporting an average of 100 students, who were teaching remotely for 5 + hours per day and who did not have their own desk/workspace to teach from home. Overall, teachers reported having structured school days, were in regular contact with their students, colleagues and parents/carers and felt they had received adequate resources and training with which to successfully teach online. However, only half of them felt their students were actively engaged in online learning or were coping well. Student engagement in remote learning is a substantial challenge. Government guidelines suggest that engagement may be enhanced by ensuring regular communication with students, using a consistent digital platform for teaching and providing school laptops and/or printed resources for students experiencing difficulties accessing technology [42].

### Mental wellbeing of teachers

The average WEMWBS score for all teachers in the current sample was 46.27. This is lower than published pre-pandemic national indicators expected for the adult general population (mean = 49.9 [43]). International findings exploring teachers’ mental wellbeing have indeed suggested that teachers mental wellbeing has been negatively affected during the pandemic [44–46]. However, the cross-sectional nature of such studies mean that it remains difficult to determine the true cause and effect of the pandemic on mental wellbeing or analyse findings over a period of time. Interestingly, however, the mean WEMWBS score for teachers in the current sample was found to be higher than that reported in the 2019 Teacher Wellbeing Index [47] for *educational professionals* in the West Midlands (mean = 43.6). This suggests that the majority of teachers who participated in our survey found the circumstances of the pandemic, including the challenges of teaching from home, had improved their mental wellbeing. All felt supported by friends or colleagues and almost two thirds felt supported by senior management and 72% by colleagues with regard to their mental wellbeing. These findings are in contrast with the Teacher Wellbeing Index in 2019 which reported that 43% of teachers, at that time, did not believe that their institutions properly supported employees who experienced problems with their mental wellbeing.

Below average mental wellbeing is indicative of mental health issues like depression and anxiety and is typically expected in 15% of the general population [41, 48]. The annual incidence of mental health issues in teachers, however, is 34% [47]. One third of teachers in our survey reported below average mental wellbeing, in keeping with pre-pandemic statistics and in line with those from our own teacher survey in 2017 [28] which showed that, pre-lockdown, teachers reported experiencing high levels of work-related stress, often due to time pressures and excessive workloads. Many failed to seek help due to stigmatic attitudes and fear of negative response by senior management and were failing to cope with extra responsibilities such as student mental wellbeing support. Some had considered leaving the teaching profession altogether, were using alcohol and tobacco to cope with stress, with only a small minority accessing psychological therapies for their problems.

The key stressors for teachers identified in the present survey were access to resources and perceived confidence to teach from home. Our findings suggest that these factors may be closely linked. Schools will have needed to adapt swiftly to the unique demands of the pandemic and, in the initial stages of school closure, may have had limited knowledge or appreciation of the necessary and appropriate technological resources and training required to enable their staff to deliver high quality remote teaching. It may be, therefore, that the confidence and capability of some teachers in the early stages of the pandemic was undermined by lack of training and support or that those who reported a lack of confidence were already struggling *before* they began remote teaching. Nonetheless, such findings suggest that school preparedness and timely provision of resources for teaching staff may have had a significant bearing on teacher confidence and efficacy, thereby impacting on their mental wellbeing. School disruptions have now been with us for some time, and, while frustrating for all involved, they will have offered a unique opportunity for schools to acquire new skills and strategies regarding the management and implementation of remote teaching. Lessons learned may help engender more positive mental wellbeing in teaching staff should they be required to step-up to similar challenges in the future.

### Practicalities of supporting learning from home for parents/carers

Over half of parent/carers said they had a structured home-schooling day, had been provided with the necessary resources and felt confident and capable in supporting their child’s learning from home. The majority of parents/carers who took part in the survey were supporting only one child with their learning from home and this was often shared with a partner. Shared responsibility may have enabled a more equal distribution of responsibilities within the family, and whilst we cannot conclude this from our data, this may have reduced individual burden and increased parents/carers’ confidence and capability in supporting learning from home. These positive responses may also be a reflection of enhanced quality time spent between a parent/carer and adolescent during lockdown. Indeed, it is believed that the more parents/carers are actively involved in the education of their child, the more likely they are to succeed in the education system [49]. Our findings should be treated with caution, however. Parent/carers schooling more than one child from home, especially single parent/carers without the support of a partner may have a different story to tell with regard to supporting learning from home.

Over one third of parents/carers reported that their child was positively engaged in learning from home and that their relationship with their child had improved during this time. However, the majority were experiencing difficulties with engagement. Encouraging self-motivation is a significant part of the educational process, especially during adolescence, much of which is heightened by a sense of school membership which can enhance motivation [50]. Remote learning practice may have impacted on this sense of membership. Lessons may have become ‘lectures’ and influenced how intrinsically motivated and engaged an adolescent was.

Schools and teachers may have strived hard to maintain engagement and sense of school membership during school closures, for example, by setting regular deadlines, providing ongoing feedback and encouraging active participation during remote teaching sessions. However, families are fundamental in ensuring that these efforts are supported and reinforced at home. Parental engagement is an on-going challenge and schools are continually searching for ways to improve their relationships with families. A range of factors will impact on parental engagement including socio-economic status and education, but this challenge will have become magnified during the pandemic [51]. The level and severity of burden and stress for home-working parents/carers, in particular, will likely have determined level of parental engagement and have varied depending on the individual needs and profiles of families. However, our findings suggest a perceived lack of support for home-working parent/carers to help reduce their burden. Burden may have been reduced significantly if schools had implemented a ‘needs-led’ approach to home learning, and, rather than operating a ‘one size fits all’ strategy, devised ways of working collaboratively with families, to help reduce inequality, support parental mental wellbeing and better understand their needs and tailor support during the pandemic.

### Mental wellbeing of parents/carers

Mean WEMWBS score for all parents/carers in the current sample was 44.01 which is markedly lower than published pre-pandemic national indicators for the adult general population (mean = 49.9 [43]). The observed difference is unlikely to be due to self-selection of a high-risk sample because participants tended to have only one child in home schooling and to have partner support. It is more likely due to a combination of factors relating to the significant disruption caused by the current pandemic. Indeed, research suggests a decrease in mental wellbeing when compared to 2019 levels, particularly in young people, women and those living in deprived areas [52]. Parents/carers who were teachers by profession fared better than those who were not. This is likely to reflect the presence of pre-existing strategies, skills and confidence of parent/carer teaching staff or that they were less worried about what a child needed to learn and better able to prioritize learning targets, which may have better prepared them for tackling and implementing learning from home.

### Working from home

Around 60% of the UK population are working from home due to the pandemic [14] and, whilst this could be argued to be preferable during such difficult circumstances, our survey suggests that home-working parents/carers struggled to co-ordinate this with their child’s educational and emotional needs leading to a negative impact on their mental wellbeing. Our findings support previous research which reports a link between level of caregiver burden and poor mental wellbeing [16]. The increase in burden may not only have impacted on parents’ mental wellbeing but also their parenting behaviours [17, 18] and their children’s cognitive and socioemotional development [53–55].

### Mental wellbeing support

Poor mental wellbeing was also observed in those parents/carers who had little or no support. Research suggests greater levels of stress in single parent/carers supporting learning from home during the pandemic compared with those living with a partner [56]. Previous studies exploring the impact of past pandemics have also shown a significant association between lack of social support and severity of mental health problems [57, 58]. Social support, whether *perceived* or *actively received*, can serve to operate as a powerful buffer, protecting individuals against the harmful impact of stressful situations [59]. There is little doubt that the COVID-19 pandemic has caused immense worry and fear [60] at a time when, paradoxically, social boundaries have been amplified and interpersonal connections and access to social support has been restricted. Problematic and extended social boundaries require innovative solutions. Greater provision and utilisation of digital social support resources, in the absence of face-to-face contact and/or therapeutic care, may have offered much needed succour for families, during this time. However, digital resources are not a ‘cure-all’ and do not address issues such as loneliness and isolation during these challenging times.

### Child-related concerns

Parents/carers whose child had little or no access to electronic devices were also observed to have poorer mental wellbeing. This may be a marker of disadvantage, however, and not solely associated with restricted access. Children and young people from higher income families have been found to receive greater support with remote learning compared to those from poorer families; higher income families devoting 30% more time to home learning and having better access to a range of remote learning resources [61]. Insufficient provision of electronic devices for students has been highlighted by The Children’s Commissioner for England who report that, in August 2020, around 9% of families were struggling with access to technology [62]. The Department for Education is now working in collaboration with several mobile network providers to help ensure that all children and young people have sufficient internet access and, to date, have provided in excess of 1.3 million disadvantaged young people and children with a laptop or tablet to enable them to fully engage with remote learning [63].

COVID related worries, difficulties with remote learning and family conflict during the pandemic have all been suggested to impact on adolescent mental health [8], with greater impact felt by those already experiencing difficulties [9]. 80% of young people with a pre-existing mental health issue believe the pandemic had made their mental health condition worse [10]. Our findings revealed that those parents/carers who felt that their child’s mental health had been affected by learning from home were also more likely to have poor mental health wellbeing. Such child-related concerns are not unexpected. Families are intrinsically motivated to attend to their child’s physical and mental wellbeing but may often lack the capacity, knowledge and awareness to do so [64]. This may lead to increased feelings of powerlessness and high levels of anxiety and stress; parental mental illness is the strongest predictor of child mental illness [65].

An additional dimension of Survey 1 was offering respondents a ‘free-text’ question *(“How has your own mental health and wellbeing been affected by supporting your child with learning from home?”).* 204 (62%) parents/carers and 52 (44%) teachers reported it had had a negative impact on their mental wellbeing. Such responses concur with our findings of poor mental wellbeing, particularly in parents/carers supporting learning and teaching from home. Given the qualitative nature of these responses, however, we were unable to include analysis of these in the present paper and they will form the basis of a separate qualitative paper.

### Limitations

Despite our aspiration of recruiting a diverse population, we were unable to translate the survey into other languages due to limited resources. This meant that the majority of respondents were of white heritage, female and from the West Midlands area. The gender and cultural bias found in the present study may be a reflection of that observed in typical survey completion. Whilst women and those of white heritage are more likely to participate in surveys [66–70] the pandemic has highlighted an important gender divide, with women taking most responsibility for childcare; carrying out an average of two-thirds more per day than men [71]. Survey topic relevance [68], and response burden [72] may have also played a role in determining those who took part, especially during such stressful circumstances. The survey was ‘live’ for a limited period, so our data only reflects a *snapshot* of the experiences of our particular sample of teachers, parents/carers during lockdown in England and their specific views *at that time*. Given the small sample size of teachers who responded to the survey, our findings related to teacher mental wellbeing should be treated with caution. Furthermore, there may be bias in our findings; teachers with a particularly positive outlook on teaching from home may have been more likely to respond. Similarly, parents/carers who found supporting their child at home *especially* difficult may be over-represented in our survey data. It is also possible that our survey may have missed key social and economic wellbeing factors that may have impacted on the experience of supporting teaching and learning from home. Given these limitations, our findings should not be generalised to the wider population and should be interpreted with caution. They are not an indication of the effects of supporting and teaching from home *before and after* the COVID-19 pandemic.

Nevertheless, the successful recruitment of 329 parents/carers and 117 teachers has provided us with useful preliminary insights into their experiences of teaching and learning from home. Such findings will serve as a foundation for further exploration and research associated with the COVID-19 pandemic.

## Conclusion

Whilst the long-term impacts of the COVID-19 pandemic are still not fully known it is predicted that there are likely to be profound physical and emotional consequences worldwide [73–75]. Findings from the COVID-19 web survey (part of the UK Household Longitudinal Study) reported a particularly marked deterioration in the mental wellbeing of women and adolescents in April 2020, compared with pre-COVID-19 trends; those living with young children and who were in employment at the beginning of the pandemic also at greater risk of mental ill health [76].

Our findings have highlighted the practical issues and challenges faced by families and teachers supporting learning from home during school closures and the factors that may have impacted on their mental wellbeing. Unexpectedly, they suggest that teacher’s mental wellbeing may not only have been protected but may have been heightened during the pandemic. Indeed, despite the challenges of the pandemic, ongoing interviews with teachers have revealed that some experience a sense of pride in being able to rise to the challenge of remote learning and appreciated the extra time, flexibility and autonomy that remote teaching had given them [77]. Recent research has found that despite the challenges of remote learning, some teachers responded very positively to the pandemic challenge. Many were willing and confident to explore and initiate a variety of new ways of working, despite their inexperience ‘in the field’ [78]. On the other hand, our findings revealed low levels of mental wellbeing in parents/carers, particularly those who were not teachers by profession. Home-schooling factors could be partly responsible for this, including a lack of knowledge, access to resources, confidence, and available time. Only half of teachers believed their students were actively engaged with on-line learning and coping well with home schooling and only a third of parents thought this was true of their adolescent. The results of this COV-ED survey will supplement existing and ongoing research findings regarding the impact of supporting learning from home during the pandemic [79, 80] and help inform schools and educational policymakers to better understand the potentially malleable factors at their disposal. This knowledge and awareness may help improve the mental wellbeing of the parents/carers and teachers asked to ‘step-up’ to the challenge of remote learning and home teaching of adolescents during the pandemic.

## Supplementary Information

**Additional file 1.**

## Data Availability

The datasets used and/or analysed during the current study are available from the corresponding author on reasonable request.

## Abbreviations

COV-ED: Coronavirus Education
UNESCO: United Nations Educational, Scientific and Cultural Organization
NASUWT: National Association of Schoolmasters and Union of Women Teachers
IPBES: Intergovernmental Science-Policy Platform on Biodiversity & Eco System Services
WEMWBS: Warwick-Edinburgh Mental Wellbeing Scale
WCTU: Warwick Clinical Trials Unit
