# Supplementary material for: The COV-ED Survey: exploring the impact of learning and teaching from home on parent/carers’ and teachers’ mental health and wellbeing during COVID-19 lockdown
Source: BMC Public Health. 2022 May 4;22:889. doi: 10.1186/s12889-022-13305-7 (PMC9066392; doi:10.1186/s12889-022-13305-7)
Supplement: Supplementary file 1 — Additional file 1. [file 12889_2022_13305_MOESM1_ESM.docx]

**Additional File 1**

**Supplementary Table 1: Teachers survey questions**

| **Section** | **Number** | **Question** | **Answer options** |
| --- | --- | --- | --- |
| **General demographics** | 1 | What is your gender? | Female, Male, Non-binary, Not listed (please specify), Prefer not to answer |
|  | 2 | What is your age? | 18-24, 25-34, 35-44, 45-54, 55-64, 65 & over, Prefer not to answer |
|  | 3 | What is your ethnicity? | Asian/Asian British, Black/African/Caribbean/Black British, Mixed ethnic background, White, Other ethnic group (please specify), Prefer not to answer |
|  | 4 | Are you a | Parent/Carer, Teacher, Both |
| *[If ‘Both’ for Q4, participant completes teacher survey first and then presented with parent/carer survey.]* | | | |
| **Teacher Introduction** | 1 | Which region is your school in? | East of England, East Midlands, London, North East, North West, South East, South West, West Midlands, Yorkshire & the Humber, Prefer not to answer |
|  | 2 | What setting is your school in? | City, Town, Village, Rural, Prefer not to answer |
|  | 3 | How would you describe your school? | Academy, Faith, Grammar, Independent/Private, Special, State/Maintained, Other (please specify), Prefer not to answer |
|  | 4 | Are you teaching from home? | Yes/No |
| *[If ‘No’ to Q4 then end survey.]* | | | |
| **Teacher Logistics** | 1 | How many students are you responsible for teaching? | Free text |
|  | 2 | What school years are you responsible for? | 7, 8, 9, 10, 11, Prefer not to answer |
|  | 3 | Are your students | Male, Female, Mixed |
|  | 4 | On average, how many hours per day are you engaged in online teaching from home? | 1, 2, 3, 4, 5 or more |
|  | 5 | This is approximately the same number of hours I am typically engaged in teaching. | Yes/No |
|  | 6 | How often do you have direct online contact with your students? | Daily, Weekly, Fortnightly, Monthly, None |
|  | 7 | I am setting regular deadlines for my students. | Yes/No |
|  |  | If yes, how often are you setting deadlines for your students? | Daily, Weekly, Fortnightly, Monthly |
|  | 8 | I am in contact with the parents/carers of my students. | Yes/No |
|  |  | If yes, how often are you in contact with the parents/carers of your students? | Daily, Weekly, Fortnightly, Monthly |
|  | 9 | I have regular contact with management at my school with regard to home teaching. | Yes/No |
|  |  | If yes, how often are you in contact with management at your school? | Daily, Weekly, Fortnightly, Monthly |
|  | 10 | I have regular contact with colleagues at my school with regard to home teaching. | Yes/No |
|  |  | If yes, how often are you in contact with colleagues at your school? | Daily, Weekly, Fortnightly, Monthly |
| *Please select how much you agree or disagree with each of the following statements.* | | | |
| **Teacher Experience** | 1 | I have a structured home teaching day. | Strongly agree, agree, neutral, disagree, strongly disagree |
|  | 2 | My school has provided me with adequate resources to teach from home. | Strongly agree, agree, neutral, disagree, strongly disagree |
|  | 3 | My school has provided me with adequate training to teach from home. | Strongly agree, agree, neutral, disagree, strongly disagree |
|  | 4 | I have my own desk and workspace to teach from home. | Strongly agree, agree, neutral, disagree, strongly disagree |
|  | 5 | I have access to a laptop/computer/tablet/smartphone to teach from home. | Strongly agree, agree, neutral, disagree, strongly disagree |
|  | 6 | I have access to the internet. | Strongly agree, agree, neutral, disagree, strongly disagree |
|  | 7 | I am not experiencing any technological difficulties when teaching from home. | Strongly agree, agree, neutral, disagree, strongly disagree |
|  | 8 | My students are engaged in online teaching. | Strongly agree, agree, neutral, disagree, strongly disagree |
|  | 9 | My students are coping well with online teaching. | Strongly agree, agree, neutral, disagree, strongly disagree |
|  | 10 | I feel supported by the management at my school with regard to home teaching. | Strongly agree, agree, neutral, disagree, strongly disagree |
|  | 11 | I feel supported by the management at my school with regard to my mental health and well-being. | Strongly agree, agree, neutral, disagree, strongly disagree |
|  | 12 | I feel supported by colleagues at my school with regard to home teaching. | Strongly agree, agree, neutral, disagree, strongly disagree |
|  | 13 | I feel supported by colleagues at my school with regard to my mental health and well-being. | Strongly agree, agree, neutral, disagree, strongly disagree |
|  | 14 | I feel confident and capable with regard to teaching from home. | Strongly agree, agree, neutral, disagree, strongly disagree |
|  | 15 | I have adapted well to using technology to teach from home. | Strongly agree, agree, neutral, disagree, strongly disagree |
|  | 16 | How has your mental health and well-being been affected by teaching from home? | Free text |
|  | 17 | I have someone to support me with my mental health and wellbeing. | Yes/No |
|  | 18 | What coping strategies are you using to help with your mental health and wellbeing? | Free text |
| *Below are some statements about feelings and thoughts. Please tick the box that best describes your experience of each over the last 2 weeks.* | | | |
| **Warwick-Edinburgh Mental Well-Being Scale** | 1 | I’ve been feeling optimistic about the future | None of the time, Rarely, Some of the time, Often, All od the time |
|  | 2 | I’ve been feeling useful | None of the time, Rarely, Some of the time, Often, All od the time |
|  | 3 | I’ve been feeling relaxed | None of the time, Rarely, Some of the time, Often, All od the time |
|  | 4 | I’ve been feeling interested in other people | None of the time, Rarely, Some of the time, Often, All od the time |
|  | 5 | I’ve had energy to spare | None of the time, Rarely, Some of the time, Often, All od the time |
|  | 6 | I’ve been dealing with problems well | None of the time, Rarely, Some of the time, Often, All od the time |
|  | 7 | I’ve been thinking clearly | None of the time, Rarely, Some of the time, Often, All od the time |
|  | 8 | I’ve been feeling good about myself | None of the time, Rarely, Some of the time, Often, All od the time |
|  | 9 | I’ve been feeling close to other people | None of the time, Rarely, Some of the time, Often, All od the time |
|  | 10 | I’ve been feeling confident | None of the time, Rarely, Some of the time, Often, All od the time |
|  | 11 | I’ve been able to make up my own mind about things | None of the time, Rarely, Some of the time, Often, All od the time |
|  | 12 | I’ve been feeling loved | None of the time, Rarely, Some of the time, Often, All od the time |
|  | 13 | I’ve been interested in new things | None of the time, Rarely, Some of the time, Often, All od the time |
|  | 14 | I’ve been feeling cheerful | None of the time, Rarely, Some of the time, Often, All od the time |
| Thank you for taking the time to complete this survey.  A list of mental health and wellbeing support organisations can be found here [link to useful resources sheet] should you need further information or guidance. | | | |

**Supplementary Table 2: Parent/carers survey questions**

| **Section** | **Number** | **Question** | **Answer options** |
| --- | --- | --- | --- |
| **General demographics** | 1 | What is your gender? | Female, Male, Non-binary, Not listed (please specify), Prefer not to answer |
|  | 2 | What is your age? | 18-24, 25-34, 35-44, 45-54, 55-64, 65 & over, Prefer not to answer |
|  | 3 | What is your ethnicity? | Asian/Asian British, Black/African/Caribbean/Black British, Mixed ethnic background, White, Other ethnic group (please specify), Prefer not to answer |
|  | 4 | Are you a | Parent/Carer, Teacher, Both |
| *[If ‘Both’ for Q4, participant completes teacher survey first and then presented with parent/carer survey.]* | | | |
| **Parent/carer Introduction** | 1 | Which region do you live in? | East of England, East Midlands, London, North East, North West, South East, South West, West Midlands, Yorkshire & the Humber, Prefer not to answer |
|  | 2 | Where do you live? | City, Town, Village, Rural, Prefer not to answer |
|  | 3 | Are you supporting your child with learning from home during lockdown? | Yes/No |
| *[If 'No' to Q3 then end survey.]* | | | |
| **Parent/carer logistics** | 1 | How many children aged 11-15 years are you supporting with learning from home? | 1, 2, 3, 4, 5 or more |
|  | 2 | Who else is supporting your child with learning from home? | Partner, Grandparent, Other family member, Not applicable |
| *[If '1' child selected message displayed - "Please answer the following questions based on your experience of supporting your child aged 11-15 years with learning from home."]*  *[If '2/3/4/5 or more' children selected message displayed - "Please answer the following questions based on your experience of supporting each of your children aged 11-15 years with learning from home. You will be asked to complete these questions individually for each child".]*  *[The child block of the survey is repeated for each child (if multiple children selected in previous section). Lead message displayed for each repetition "Please answer the following questions based on your first/second/third/fourth/fifth child (aged 11-15 years)." If '5 or more' children selected, then survey only requests information for first five children aged 11-15 years.]* | | | |
| **Child-related** | 1 | What school year are they in? | 7, 8, 9, 10, 11, Prefer not to answer |
|  | 2 | What type of school does your child attend? | Academy, Faith, Grammar, Independent/Private, Special, State/Maintained, Other (please specify), Prefer not to answer |
|  | 3 | How many hours per day on average are you supporting your child with learning from home? | 1, 2, 3, 4, 5 or more |
|  | *Please select how much you agree or disagree with each of the following statements.* | | |
|  | 4 | I have a structured home schooling day. | Strongly agree, agree, neutral, disagree, strongly disagree |
|  | 5 | My child’s school has provided me with adequate resources to use for learning from home. | Strongly agree, agree, neutral, disagree, strongly disagree |
|  | 6 | I am using other resources for learning from home. | Strongly agree, agree, neutral, disagree, strongly disagree |
|  | 7 | My child has access to their own laptop/computer/tablet/smartphone to complete school work. | Strongly agree, agree, neutral, disagree, strongly disagree |
|  | 8 | My child has access to the internet. | Strongly agree, agree, neutral, disagree, strongly disagree |
|  | 9 | My child has access to their own desk and workspace for school work. | Strongly agree, agree, neutral, disagree, strongly disagree |
|  | 10 | I am also working from home at the same time. | Strongly agree, agree, neutral, disagree, strongly disagree |
|  | 11 | Working from home at the same time as supporting my child with learning is easy to coordinate. | Strongly agree, agree, neutral, disagree, strongly disagree |
|  | 12 | I am having regular contact with my child’s school and/or teacher. | Strongly agree, agree, neutral, disagree, strongly disagree |
|  | 13 | I feel supported by my child’s school and/or teacher and know who to contact if I have any problems with learning from home. | Strongly agree, agree, neutral, disagree, strongly disagree |
|  | 14 | My child’s school and/or teacher has set deadlines for my child’s work. | Strongly agree, agree, neutral, disagree, strongly disagree |
|  | 15 | I feel capable and confident in supporting my child with learning from home. | Strongly agree, agree, neutral, disagree, strongly disagree |
|  | 16 | I feel capable and confident using technology to support my child with learning from home. | Strongly agree, agree, neutral, disagree, strongly disagree |
|  | 17 | My child is engaged with and enjoying learning from home. | Strongly agree, agree, neutral, disagree, strongly disagree |
|  | 18 | My relationship with my child has improved whilst learning from home. | Strongly agree, agree, neutral, disagree, strongly disagree |
|  | 19 | My child’s mental health and wellbeing has been affected by learning from home. | Yes/No |
|  |  | If yes, in what way has your child’s mental health and wellbeing been affected by learning from home? | Free text |
| **Parent experience** | 1 | How has your own mental health and wellbeing been affected by supporting your child with learning from home? | Free text |
|  | 2 | I have someone to support me with my mental health and wellbeing. | Yes/No |
|  | 3 | What coping strategies are you using to help you maintain your mental health and emotional wellbeing? | Free text |
| *Below are some statements about feelings and thoughts. Please tick the box that best describes your experience of each over the last 2 weeks.* | | | |
| **Warwick-Edinburgh Mental Well-Being Scales** | 1 | I’ve been feeling optimistic about the future | None of the time, Rarely, Some of the time, Often, All od the time |
|  | 2 | I’ve been feeling useful | None of the time, Rarely, Some of the time, Often, All od the time |
|  | 3 | I’ve been feeling relaxed | None of the time, Rarely, Some of the time, Often, All od the time |
|  | 4 | I’ve been feeling interested in other people | None of the time, Rarely, Some of the time, Often, All od the time |
|  | 5 | I’ve had energy to spare | None of the time, Rarely, Some of the time, Often, All od the time |
|  | 6 | I’ve been dealing with problems well | None of the time, Rarely, Some of the time, Often, All od the time |
|  | 7 | I’ve been thinking clearly | None of the time, Rarely, Some of the time, Often, All od the time |
|  | 8 | I’ve been feeling good about myself | None of the time, Rarely, Some of the time, Often, All od the time |
|  | 9 | I’ve been feeling close to other people | None of the time, Rarely, Some of the time, Often, All od the time |
|  | 10 | I’ve been feeling confident | None of the time, Rarely, Some of the time, Often, All od the time |
|  | 11 | I’ve been able to make up my own mind about things | None of the time, Rarely, Some of the time, Often, All od the time |
|  | 12 | I’ve been feeling loved | None of the time, Rarely, Some of the time, Often, All od the time |
|  | 13 | I’ve been interested in new things | None of the time, Rarely, Some of the time, Often, All od the time |
|  | 14 | I’ve been feeling cheerful | None of the time, Rarely, Some of the time, Often, All od the time |
| Thank you for taking the time to complete this survey.  A list of mental health and wellbeing support organisations can be found here [link to useful resources sheet] should you need further information or guidance. | | | |

**Supplementary Table 3: Details of questions combined for inclusion in regression models**

| **Teachers’ survey questions** | **Question** | **Likert scale** | **Coding** | **Combined measure** |
| --- | --- | --- | --- | --- |
| Teacher experience  (Q10) | I feel supported by the management at my school with regard to home teaching | Strongly agree, agree, neutral, disagree, strongly disagree. | 1-5 respectively | School support = question 1 + question 2  Scores range from 2-10  Categorised as follows: 2-3 = high support, 4 = moderate support, 5-1 0= low support. |
| Teacher experience (Q11) | I feel supported by the management at my school with regard to my mental health and well-being | Strongly agree, agree, neutral, disagree, strongly disagree | 1-5 respectively |  |
| Teacher experience (Q12) | I feel supported by colleagues at my school with regard to home teaching. | Strongly agree, agree, neutral, disagree, strongly disagree. | 1-5 respectively | Colleague support = question 3 + question 4  Scores range from 2-10  Categorised as follows: 2-3 = high support, 4 = moderate support, 5-10 = low support. |
| Teacher experience (Q13) | I feel supported by colleagues at my school with regard to my mental health and well-being. | Strongly agree, agree, neutral, disagree, strongly disagree | 1-5 respectively |  |

**Supplementary Table 4: The practicalities of teaching from home for teachers**

|  | **Teachers that aren’t parents**  **N=76** | **Teachers that are parents**  **N=41** | **All teachers**  **N=117** |
| --- | --- | --- | --- |
| **How many students are you responsible for teaching?**  **(median, iqr)** | 118 (67-197) | 50 (15-120) | 100 (44-165) |
| **Student gender** |  |  |  |
| Male | 5 (6.6%) | 7 (17.1%) | 12 (10.3%) |
| Female | 14 (18.4%) | 5 (12.2%) | 19 (16.2%) |
| Mixed | 57 (75%) | 29 (70.7%) | 86 (73.5%) |
| **On average, how many hours per day are you engaged in online teaching from home?** |  |  |  |
| 1 | 11 (14.5%) | 4 (9.8%) | 15 (12.8%) |
| 2 | 14 (18.4%) | 9 (22.0%) | 23 (19.7%) |
| 3 | 15 (19.7%) | 8 (19.5%) | 23 (19.7%) |
| 4 | 7 (9.2%) | 7 (17.1%) | 14 (12.0%) |
| 5 or more | 29 (38.2%) | 13 (31.7%) | 42 (35.9%) |
| **How often do you have direct online contact with your students?** |  |  |  |
| Daily | 40 (52.6%) | 23 (56.1%) | 63 (53.8%) |
| Weekly | 30 (39.5%) | 14 (34.1%) | 44 (37.6%) |
| Fortnightly | 1 (1.3%) | 0 (0.0%) | 1 (0.9%) |
| Monthly |  |  |  |
| None | 5 (6.6%) | 4 (9.8%) | 9 (7.7%) |
| **I am setting regular deadlines for my students.** |  |  |  |
| Yes | 70 (92.1%) | 34 (82.9%) | 104 (88.9%) |
| No | 6 (7.9%) | 7 (17.1%) | 13 (11.1%) |
| **I am in contact with the parents/carers of my students** |  |  |  |
| Yes | 58 (76.3%) | 29 (70.7%) | 87 (74.4%) |
| No | 18 (23.7%) | 12 (29.3%) | 30 (25.6%) |
| **I have regular contact with management at my school with regard to home teaching** |  |  |  |
| Yes | 71 (93.4%) | 37 (90.2%) | 108 (92.3%) |
| No | 4 (5.3%) | 4 (9.8%) | 8 (6.8%) |
| Unknown | 1 (1.3%) | 0 (0.0%) | 1 (0.9%) |
| **I have regular contact with colleagues at my school with regard to home teaching** |  |  |  |
| Yes | 72 (94.7%) | 36 (87.8%) | 108 (92.3%) |
| No | 4 (5.3%) | 5 (12.2%) | 9 (7.7%) |
| **I have a structured home teaching day.** |  |  |  |
| Strongly agree | 23 (30.3%) | 11 (26.8%) | 34 (29.1%) |
| Agree | 29 (38.2%) | 17 (41.5%) | 46 (39.3%) |
| Neutral | 11 (14.5%) | 4 (9.8%) | 15 (12.8%) |
| Disagree | 9 (11.8%) | 5 (12.2%) | 14 (12.0%) |
| Strongly disagree | 4 (5.3%) | 3 (7.3%) | 7 (6.0%) |
| Unknown |  | 1 (2.4%) | 1 (0.9%) |
| **My school has provided me with adequate resources to teach from home.** |  |  |  |
| Strongly agree | 24 (31.6%) | 11 (26.8%) | 35 (29.9%) |
| Agree | 32 (42.1%) | 14 (34.2%) | 46 (39.3%) |
| Neutral | 13 (17.1%) | 9 (22.0%) | 22 (18.8%) |
| Disagree | 3 (4.0%) | 5 (12.2%) | 8 (6.8%) |
| Strongly disagree | 4 (5.3%) | 2 (4.9%) | 6 (5.1%) |
| **My school has provided me with adequate training to teach from home.** |  |  |  |
| Strongly agree | 21 (27.6%) | 9 (22.0%) | 30 (25.6%) |
| Agree | 32 (42.1%) | 11 (26.8%) | 43 (36.8%) |
| Neutral | 11 (14.5%) | 10 (24.4%) | 21 (18.0%) |
| Disagree | 9 (11.8%) | 7 (17.1%) | 16 (13.7%) |
| Strongly disagree | 3 (4.0%) | 4 (9.8%) | 7 (6.0%) |
| **I have my own desk and workspace to teach from home.** |  |  |  |
| Strongly agree | 20 (26.3%) | 10 (24.4%) | 30 (25.6%) |
| Agree | 18 (23.7%) | 10 (24.4%) | 28 (23.9%) |
| Neutral | 4 (5.3%) | 6 (14.6%) | 10 (8.6%) |
| Disagree | 25 (32.9%) | 13 (31.7%) | 38 (32.5%) |
| Strongly disagree | 9 (11.8%) | 2 (4.9%) | 11 (9.4%) |
| **I have access to a laptop/computer/tablet/smartphone to teach from home.** |  |  |  |
| Strongly agree | 53 (69.7%) | 27 (65.9%) | 80 (68.4%) |
| Agree | 22 (29.0%) | 13 (31.7%) | 35 (29.9%) |
| Neutral | 1 (1.3%) | 1 (2.4%) | 2 (1.7%) |
| Disagree | 0 (0.0%) | 0 (0.0%) | 0 (0.0%) |
| Strongly disagree | 0 (0.0%) | 0 (0.0%) | 0 (0.0%) |
| **I have access to the internet.** |  |  |  |
| Strongly agree | 53 (69.7%) | 27 (65.9%) | 80 (68.4%) |
| Agree | 21 (27.6%) | 14 (34.2%) | 35 (29.9%) |
| Neutral | 1 (1.3%) | 0 (0.0%) | 1 (0.9%) |
| Disagree | 0 (0.0%) | 0 (0.0%) | 0 (0.0%) |
| Strongly disagree | 1 (1.3%) | 0 (0.0%) | 1 (0.8%) |
| **I am not experiencing any technological difficulties when teaching from home** |  |  |  |
| Strongly agree | 15 (19.7%) | 5 (12.2%) | 20 (17.1%) |
| Agree | 32 (42.1%) | 12 (29.3%) | 44 (37.6%) |
| Neutral | 14 (18.4%) | 13 (31.7%) | 27 (23.1%) |
| Disagree | 13 (17.1%) | 9 (22.0%) | 22 (18.8%) |
| Strongly disagree | 2 (2.6%) | 2 (4.9%) | 4 (3.4%) |
| **My students are engaged in online teaching.** |  |  |  |
| Strongly agree | 12 (15.8%) | 3 (7.3%) | 15 (12.8%) |
| Agree | 34 (44 7%) | 12 (29.3%) | 46 (39.3%) |
| Neutral | 13 (17.1%) | 16 (39.0%) | 29 (24.8%) |
| Disagree | 12 (15.8%) | 8 (19.5%) | 20 (17.1%) |
| Strongly disagree | 5 (6.6%) | 2 (4.9%) | 7 (6.0%) |
| **My students are coping well with online teaching.** |  |  |  |
| Strongly agree | 7 (9.7%) | 3 (7.3%) | 10 (8.6%) |
| Agree | 34 (44.7%) | 15 (36.6%) | 49 (41.9%) |
| Neutral | 23 (30.3%) | 12 (29.3%) | 35 (29.9%) |
| Disagree | 7 (9.2%) | 11 (26.8%) | 18 (15.4%) |
| Strongly disagree | 5 (6.6%) | 0 (0.0%) | 5 (4.3%) |
| **I feel supported by the management at my school with regard to home teaching** |  |  |  |
| Strongly agree | 25 (32.9%) | 11 (26.8%) | 36 (30.8%) |
| Agree | 37 (48.7%) | 16 (39.0%) | 53 (45.3%) |
| Neutral | 5 (6.6%) | 6 (14.6%) | 11 (9.4%) |
| Disagree | 6 (7.9%) | 7 (17.1%) | 13 (11.1%) |
| Strongly disagree | 3 (4.0%) | 1 (2.4%) | 4 (3.4%) |
| **I feel supported by the management at my school with regard to my mental health and well-being** |  |  |  |
| Strongly agree | 15 (19.7%) | 8 (19.5%) | 23 (19.7%) |
| Agree | 34 (44.7%) | 16 (39.0%) | 50 (42.7%) |
| Neutral | 12 (15.8%) | 7 (17.1%) | 19 (16.2%) |
| Disagree | 8 (10.5%) | 6 (14.6%) | 14 (12.0%) |
| Strongly disagree | 7 (9.2%) | 4 (9.8%) | 11 (9.4%) |
| **I feel supported by colleagues at my school with regard to home teaching** |  |  |  |
| Strongly agree | 24 (31.6%) | 13 (31.7%) | 37 (31.6%) |
| Agree | 42 (55.3%) | 20 (48.8%) | 62 (53.0%) |
| Neutral | 4 (5.3%) | 5 (12.2%) | 9 (7.7%) |
| Disagree | 5 (6.6%) | 2 (4.9%) | 7 (6.0%) |
| Strongly disagree | 1 (1.3%) | 0 (0.0%) | 1 (0.8%) |
| Unknown | 0 (0.0%) | 1 (2.4%) | 1 (0.9%) |
| **I feel supported by colleagues at my school with regard to my mental health and well-being** |  |  |  |
| Strongly agree | 18 (23.7%) | 11 (26.8%) | 29 (24.8%) |
| Agree | 34 (44.7%) | 21 (51.2%) | 55 (47.0%) |
| Neutral | 16 (21.1%) | 3 (7.3%) | 19 (16.2%) |
| Disagree | 4 (5.3%) | 5 (12.2%) | 9 (7.7%) |
| Strongly disagree | 3 (4.0%) | 1 (2.4%) | 4 (3.4%) |
| Unknown | 1 (1.3%) | 0 (0.0%) | 1 (0.9) |
| **I feel confident and capable with regard to teaching from home.** |  |  |  |
| Strongly agree | 24 (31.6%) | 11 (26.8%) | 35 (29.9%) |
| Agree | 40 (52.6%) | 17 (41.5%) | 57 (48.7%) |
| Neutral | 10 (13.2%) | 7 (17.1%) | 17 (14.5%) |
| Disagree | 1 (1.3%) | 5 (12.2%) | 6 (5.1%) |
| Strongly disagree | 1 (1.3%) | 1 (2.4%) | 2 (1.7%) |
| **I have adapted well to using technology to teach from home.** |  |  |  |
| Strongly agree | 24 (31.6%) | 12 (29.3%) | 36 (30.8%) |
| Agree | 44 (57.9%) | 21 (51.2%) | 65 (55.6%) |
| Neutral | 6 (7.9%) | 4 (9.8%) | 10 (8.9%) |
| Disagree | 1 (1.3%) | 3 (7.3%) | 4 (3.4%) |
| Strongly disagree | 1 (1.3%) | 0 (0.0%) | 1 (0.9%) |
| Unknown | 0 (0.0%) | 1 (2.4%) | 1 (0.8%) |
| **I have someone to support me with my mental health and wellbeing.** |  |  |  |
| Yes | 65 (85.5%) | 31 (75.6%) | 96 (82.1%) |
| No | 11 (14.5%) | 10 (24.4%) | 21 (17.9%) |

**Supplementary Table 5a: The practicalities of supporting a child/children with learning from home (family level)**

|  | **Parents that aren’t teachers**  **N=284** | **Parents that are teachers**  **N=45** | **All parents**  **N=329** |
| --- | --- | --- | --- |
| **How many children aged 11-15 years are you supporting with learning from home?** |  |  |  |
| 1 | 197 (69.4%) | 28 (62.2%) | 225 (68.4%) |
| 2 | 81 (28.5%) | 17 (37.8%) | 98 (29.8%) |
| 3 | 6 (2.1%) | 0 (0.0%) | 6 (1.8%) |
| **Who else is supporting your child with learning from home?** |  |  |  |
| Partner | 178 (62.7%) | 26 (57.8%) | 204 (62.0%) |
| Grandparent | 4 (1.4%) | 2 (4.4%) | 6 (1.8%) |
| Other family member | 17 (6.0%) | 1 (2.2%) | 18 (5.5%) |
| Not applicable | 85 (29.9%) | 16 (35.6%) | 101 (30.7%) |
| **I have someone to support me with my mental health and wellbeing.** |  |  |  |
| Yes | 175 (61.6%) | 34 (75.6%) | 209 (63.5%) |
| No | 107 (37.7%) | 11 (24.4%) | 118 (35.9%) |
| Unknown | 2 (0.7%) | 0 (0.0%) | 2 (0.6%) |

**Supplementary Table 5b: The practicalities of supporting a child/children with learning from home for parents/carers (child-level)**

| **Child–related:** | **Child 1**  **N=329** | **Child 2**  **N=104** | **Child 3**  **N=6** |
| --- | --- | --- | --- |
| **What year is your child in?** |  |  |  |
| 7 | 91 (27.7%) | 28 (26.9%) | 2 (33.3%) |
| 8 | 78 (23.7%) | 22 (21.2%) | 0 (0%) |
| 9 | 73 (22.2%) | 19 (18.3%) | 0 (0%) |
| 10 | 61 (18.5%) | 18 (17.3%) | 0 (0%) |
| 11 | 15 (4.6%) | 6 (5.8%) | 2 (33.3%) |
| Unknown | 11 (3.3%) | 11 (10.6%) | 2 (33.3%) |
| **What type of school does your child attend?** |  |  |  |
| Academy | 150 (45.6%) | 43 (41.4%) | 2 (33.3%) |
| Faith | 36 (10.9%) | 11 (10.6%) | 1 (16.7%) |
| Grammar | 22 (6.7%) | 8 (7.7%) | 0 (0%) |
| Independent/private | 43 (13.1%) | 13 (12.5%) | 0 (0%) |
| Special | 11 (3.3%) | 0 (0%) | 0 (0%) |
| State/maintained | 59 (17.9%) | 23 (22.1%) | 3 (50%) |
| Other | 8 (2.4%) | 6 (5.8%) | 0 (0%) |
| **How many hours per day on average are you supporting your child with learning from home?** |  |  |  |
| 1 | 132 (40.1%) | 51 (49%) | 4 (66.7%) |
| 2 | 78 (23.7%) | 24 (23.1%) | 1 (16.7%) |
| 3 | 50 (15.2%) | 10 (9.6%) | 0 (0%) |
| 4 | 37 (11.3%) | 12 (11.5%) | 1 (16.7%) |
| 5+ | 32 (9.7%) | 7 (6.7%) | 0 (0%) |
| **I have a structured home schooling day.** |  |  |  |
| Strongly agree | 69 (21%) | 23 (22.1%) | 0 (0%) |
| Agree | 110 (33.4%) | 43 (41.4%) | 2 (33.3%) |
| Neutral | 55 (16.7%) | 15 (14.4%) | 2 (33.3%) |
| Disagree | 61 (18.5%) | 16 (15.4%) | 1 (16.7%) |
| Strongly disagree | 32 (9.7%) | 5 (4.8%) | 1 (16.7%) |
| Unknown | 2 (0.6%) | 2 (1.9%) | 0 (0%) |
| **My child’s school has provided me with adequate resources to use for learning from home** |  |  |  |
| Strongly agree | 64 (19.5%) | 21 (20.2%) | 0 (0%) |
| Agree | 127 (38.6%) | 36 (34.6%) | 0 (0%) |
| Neutral | 54 (16.4%) | 17 (16.4%) | 4 (66.7%) |
| Disagree | 62 (18.8%) | 24 (23.1%) | 0 (0%) |
| Strongly disagree | 18 (5.5%) | 4 (3.9%) | 2 (33.3%) |
| Unknown | 4 (1.2%) | 2 (1.9%) | 0 (0%) |
| **I am using other resources for learning from home** |  |  |  |
| Strongly agree | 47 (14.3%) | 10 (9.6%) | 0 (0%) |
| Agree | 144 (43.8%) | 49 (47.1%) | 4 (66.7%) |
| Neutral | 41 (12.5%) | 17 (16.4%) | 2 (33.3%) |
| Disagree | 70 (21.3%) | 16 (15.4%) | 0 (0%) |
| Strongly disagree | 22 (6.7%) | 9 (8.7%) | 0 (0%) |
| Unknown | 5 (1.5%) | 3 (2.9%) | 0 (0%) |
| **My child has access to their own laptop/computer/tablet/smartphone to complete school work.** |  |  |  |
| Strongly agree | 213 (64.7%) | 65 (62.5%) | 3 (50%) |
| Agree | 77 (23.4%) | 26 (25%) | 2 (33.3%) |
| Neutral | 9 (2.7%) | 4 (3.9%) | 1 (16.7%) |
| Disagree | 21 (6.4%) | 6 (5.8%) | 0 (0%) |
| Strongly disagree | 6 (1.8%) | 1 (1%) | 0 (0%) |
| Unknown | 3 (0.9%) | 2 (1.9%) | 0 (0%) |
| **My child has access to the internet.** |  |  |  |
| Strongly agree | 248 (75.4%) | 76 (73.1%) | 3 (50%) |
| Agree | 74 (22.5%) | 25 (24%) | 2 (33.3%) |
| Neutral | 3 (0.9%) | 1 (1%) | 1 (16.7%) |
| Disagree | 0 (0%) | 0 (0%) | 0 (0%) |
| Strongly disagree | 1 (0.3%) | 0 (0%) | 0 (0%) |
| Unknown | 3 (0.9%) | 2 (1.9%) | 0 (0%) |
| **My child has access to their own desk and workspace for school work.** |  |  |  |
| Strongly agree | 193 (58.7%) | 59 (56.7%) | 2 (33.3%) |
| Agree | 62 (18.8%) | 25 (24%) | 2 (33.3%) |
| Neutral | 19 (5.8%) | 4 (3.9%) | 1 (16.7%) |
| Disagree | 42 (12.8%) | 7 (6.7%) | 0 (0%) |
| Strongly disagree | 10 (3%) | 7 (6.7%) | 1 (16.7%) |
| Unknown | 3 (0.9%) | 2 (1.9%) | 0 (0%) |
| **I am also working from home at the same time.** |  |  |  |
| Strongly agree | 178 (54.1%) | 56 (53.9%) | 1 (16.7%) |
| Agree | 57 (17.3%) | 21 (20.2%) | 1 (16.7%) |
| Neutral | 22 (6.7%) | 7 (6.7%) | 1 (16.7%) |
| Disagree | 45 (13.7%) | 9 (8.7%) | 1 (16.7%) |
| Strongly disagree | 24 (7.3%) | 9 (8.7%) | 2 (33.3%) |
| Unknown | 3 (0.9%) | 2 (1.9%) | 0 (0%) |
| **Working from home at the same time as supporting my child with learning is easy to coordinate.** |  |  |  |
| Strongly agree | 14 (4.3%) | 8 (7.7%) | 0 (0%) |
| Agree | 44 (13.4%) | 7 (6.7%) | 2 (33.3%) |
| Neutral | 75 (22.8%) | 25 (24%) | 4 (66.7%) |
| Disagree | 102 (31%) | 35 (33.7%) | 0 (0%) |
| Strongly disagree | 90 (27.4%) | 27 (26%) | 0 (0%) |
| Unknown | 4 (1.2%) | 2 (1.9%) | 0 (0%) |
| **I am having regular contact with my child’s school and/or teacher.** |  |  |  |
| Strongly agree | 29 (8.8%) | 10 (9.6%) | 0 (0%) |
| Agree | 93 (28.3%) | 24 (23.1%) | 0 (0%) |
| Neutral | 56 (17%) | 24 (23.1%) | 4 (66.7%) |
| Disagree | 87 (26.4%) | 30 (28.9%) | 1 (16.7%) |
| Strongly disagree | 62 (18.8%) | 14 (13.5%) | 1 (16.7%) |
| Unknown | 2 (0.6%) | 2 (1.9%) | 0 (0%) |
| **I feel supported by my child’s school and/or teacher and know who to contact if I have any problems with learning from home.** |  |  |  |
| Strongly agree | 51 (15.5%) | 11 (10.6%) | 0 (0%) |
| Agree | 125 (38%) | 38 (36.5%) | 1 (16.7%) |
| Neutral | 52 (15.8%) | 24 (23.1%) | 3 (50%) |
| Disagree | 62 (18.8%) | 16 (15.4%) | 0 (0%) |
| Strongly disagree | 37 (11.3%) | 13 (12.5%) | 2 (33.3%) |
| Unknown | 2 (0.6%) | 2 (1.9%) | 0 (0%) |
| **My child’s school and/or teacher has set deadlines for my child’s work.** |  |  |  |
| Strongly agree | 84 (25.5%) | 23 (22.1%) | 0 (0%) |
| Agree | 140 (42.6%) | 54 (51.9%) | 1 (16.7%) |
| Neutral | 36 (10.9%) | 11 (10.6%) | 3 (50%) |
| Disagree | 41 (12.5%) | 10 (9.6%) | 1 (16.7%) |
| Strongly disagree | 27 (8.2%) | 4 (3.9%) | 1 (16.7%) |
| Unknown | 1 (0.3%) | 2 (1.9%) | 0 (0%) |
| **I feel capable and confident in supporting my child with learning from home.** |  |  |  |
| Strongly agree | 47 (14.3%) | 13 (12.5%) | 1 (16.7%) |
| Agree | 96 (29.2%) | 35 (33.7%) | 1 (16.7%) |
| Neutral | 75 (22.8%) | 21 (20.2%) | 3 (50%) |
| Disagree | 80 (24.3%) | 21 (20.2%) | 0 (0%) |
| Strongly disagree | 29 (8.8%) | 11 (10.6%) | 1 (16.7%) |
| Unknown | 2 (0.6%) | 3 (2.9%) | 0 (0%) |
| **I feel capable and confident using technology to support my child with learning from home.** |  |  |  |
| Strongly agree | 75 (22.8%) | 27 (26%) | 1 (16.7%) |
| Agree | 143 (43.5%) | 39 (37.5%) | 1 (16.7%) |
| Neutral | 59 (17.9%) | 19 (18.3%) | 3 (50%) |
| Disagree | 40 (12.2%) | 11 (10.6%) | 0 (0%) |
| Strongly disagree | 10 (3%) | 4 (3.9%) | 1 (16.7%) |
| Unknown | 2 (0.6%) | 4 (3.9%) | 0 (0%) |
| **My child is engaged with and enjoying learning from home.** |  |  |  |
| Strongly agree | 44 (13.4%) | 13 (12.5%) | 0 (0%) |
| Agree | 85 (25.8%) | 28 (26.9%) | 1 (16.7%) |
| Neutral | 77 (23.4%) | 27 (26%) | 2 (33.3%) |
| Disagree | 70 (21.3%) | 17 (16.4%) | 1 (16.7%) |
| Strongly disagree | 50 (15.2%) | 16 (15.4%) | 1 (16.7%) |
| Unknown | 3 (0.9%) | 3 (2.9%) | 1 (16.7%) |
| **My relationship with my child has improved whilst learning from home.** |  |  |  |
| Strongly agree | 32 (9.7%) | 9 (8.7%) | 0 (0%) |
| Agree | 81 (24.6%) | 31 (29.8%) | 2 (33.3%) |
| Neutral | 136 (41.3%) | 40 (38.5%) | 3 (50%) |
| Disagree | 56 (17%) | 17 (16.4%) | 0 (0%) |
| Strongly disagree | 22 (6.7%) | 5 (4.8%) | 1 (16.7%) |
| Unknown | 2 (0.6%) | 2 (1.9%) | 0 (0%) |
| **My child’s mental health and wellbeing has been affected by learning from home.** |  |  |  |
| No | 125 (38%) | 45 (43.3%) | 4 (66.7%) |
| Yes | 204 (62%) | 58 (55.8%) | 2 (33.3%) |
| Unknown | 0 (0%) | 1 (1%) | 0 (0%) |

**Supplementary Table 6:** **Univariable linear regression model exploring effect of teacher survey responses on teachers’ mental well-being**

In the univariable models, the survey questions “*My school has provided me with adequate resources to teach from home”,* “*I feel confident and capable with regard to teaching from home*” and “*My students are coping well with online teaching*” were found to be significantly association with the mental health and well-being of teachers (per WEMWBS score).

Compared to teachers who *strongly* agreed with the statement “*My school has provided me with adequate resources to teach from home”,* WEMWBS scores were on average 3.06 points lower in those who agreed (95% CI -6.71 – 0.59), 5.61 points lower in those who were neutral (95% CI -10.05 – -1.18) and 11.31 points lower in those who disagreed (95% CI -16.47 – -6.16) (p<0.001).

Similarly, compared to teachers who strongly agreed with the statement “*I feel confident and capable with regard to my teaching at home*”, WEMWBS scores were on average 2.08 points lower in those who agreed (95% CI -5.60 – 1.44), 3.86 points lower in those who were neutral (95% CI -8.70 – 0.98) and 13.93 points lower in those who disagreed (-20.34 – -7.51)(p<0.001).

Compared to teachers who strongly agreed with the statement *“My students are coping well with online teaching”,* WEMWBS scores were on average 1.35 points lower in those who agreed (95% CI -7.17 – 4.48), 1.77 points lower in those who were neutral (95% CI -7.79 – 4.25) and 8.30 points lower in those who disagreed (-14.66 – -1.95) (p<0.01).

|  | **Univariable model** | |
| --- | --- | --- |
| **Factor** | **Coefficient (95% CI)** | **p-value** |
| **Gender** |  |  |
| Male | Ref |  |
| Female | -2.35 (-6.28 – 1.59) | 0.24 |
| **Age** |  |  |
| Under 45 years | Ref |  |
| 45 years and over | 2.46 (-0.76 – 5.67) | 0.13 |
| **Ethnicity** |  |  |
| Minority ethnicities | Ref |  |
| White | -0.76 (-6.13 – 4.62) | 0.78 |
| **I have someone to support me with my mental health and wellbeing** |  |  |
| No | Ref |  |
| Yes | 2.31 (-1.90 – 6.51) | 0.28 |
| **Feel supported by school** |  |  |
| High (score 2-3) | Ref |  |
| Moderate (score 4) | -.89 (-5.11 – 3.34) |  |
| Low (score 5 or more) | -3.29 (-7.27 – 0.68) | 0.23 |
| **Feel supported by colleagues** |  |  |
| High (score 2-3) | Ref |  |
| Moderate (score 4) | -2.12 (-6.00 – 1.76) |  |
| Low (score 5 or more) | -3.28 (-7.54 – 0.99) | 0.30 |
| **My school has provided me with adequate resources to teach from home** |  |  |
| Strongly agree | Ref |  |
| Agree | -3.06 (-6.71 – 0.59) |  |
| Neutral | -5.61 (-10.05 – -1.18) |  |
| Disagree/Strongly disagree | -11.31 (-16.47 – -6.16) | <0.001 |
| **My school has provided me with adequate training to teach from home** |  |  |
| Strongly agree | Ref |  |
| Agree | -1.24 (-5.38 – 2.91) |  |
| Neutral | -1.65 (-6.60 – 3.31) |  |
| Disagree/Strongly disagree | -4.63 (-9.46 – 0.20) | 0.29 |
| **I have my own desk and workspace to teach from home** |  |  |
| Strongly agree | Ref |  |
| Agree | -0.07 (-4.69 – 4.55) |  |
| Neutral | 1.10 (-5.32 – 7.52) |  |
| Disagree/Strongly disagree | -1.92 (-5.99 – 2.16) | 0.65 |
| **I feel confident and capable with regard to teaching from home** |  |  |
| Strongly agree | Ref |  |
| Agree | -2.08 (-5.60 – 1.44) |  |
| Neutral | -3.86 (-8.70 – 0.98) |  |
| Disagree/Strongly disagree | -13.93 (-20.34 – -7.51) | <0.001 |
| **My students are engaged in online teaching** |  |  |
| Strongly agree | Ref |  |
| Agree | -1.36 (-6.59 – 3.87) |  |
| Neutral | -3.02 (-8.61 – 2.57) |  |
| Disagree/Strongly disagree | -3.61 (-11.66 – 4.44) | 0.62 |
| **My students are coping well with online teaching** |  |  |
| Strongly agree | Ref |  |
| Agree | -1.35 (-7.17 – 4.48) |  |
| Neutral | -1.77 (-7.79 – 4.25) |  |
| Disagree/Strongly disagree | -8.30 (-14.66 – -1.95) | <0.01 |

**Supplementary Table 7:** **Univariable linear regression model exploring effect of parent survey responses on parents’ mental well-being**

In the univariable models, answers to the following survey questions were mostly strongly associated with mental health and wellbeing in parents. Parents who disagreed with the statement *”Working from home at the same time as supporting my child with learning is easy to coordinate”,* for example, had WEMWEBS scores that were 11.16 points lower on average than parents who strongly agree (95% CI -13.93 – -8.39), indicating markedly poorer mental health and wellbeing. Similarly, parents who disagreed with the statement “*I feel supported by my child’s school and/or teacher and know who to contact if I have any problems with learning from home*” scored 10.63 fewer points on average (95% CI -13.75 – -7.51) than those who strongly agreed. Parents who disagreed with the statement *“I feel capable and confident in supporting my child with learning from home”* scored 12.25 fewer points per WEMWEBS on average (95% CI -15.31 – -9.19) and parents who disagreed with the statement *“My relationship with my child has improved whilst learning from home*” scored 12.31 fewer points (95% CI 16.23 – -8.39).

|  | **Univariable models** | |
| --- | --- | --- |
| **Factor** | **Coefficient (95% CI)** | **p-value** |
| **Gender** |  |  |
| Male | Ref |  |
| Female | -2.99 (-6.02 – 0.05) | 0.05 |
| **Age** |  |  |
| Under 45 years | Ref |  |
| 45 years and over | -0.03 (-2.30 – 2.23) | 0.98 |
| **Ethnicity** |  |  |
| Minority ethnicities | Ref |  |
| White | -2.66 (-7.75 – 2.43) | 0.30 |
| **Who else is supporting your child with learning from home?** |  |  |
| Not applicable | Ref |  |
| Parent/grandparent/other family member | 1.65 (-0.65 – 3.95) | 0.16 |
| **I have someone to support me with my mental health and wellbeing** |  |  |
| No | Ref |  |
| Yes | 5.00 (2.56 – 7.44) | <0.001 |
| **I have a structured home schooling day** |  |  |
| Strongly agree | Ref |  |
| Agree | -1.71 (-4.48 – 1.06) |  |
| Neutral | -1.68 (-5.17 – 1.82) |  |
| Disagree/strongly disagree | -6.29 (-9.26 – -3.33) | <0.001 |
| **My child’s school has provided me with adequate resources to use for learning from home** |  |  |
| Strongly agree | Ref |  |
| Agree | -4.63 (-7.34 – -1.93) |  |
| Neutral | -3.98 (-6.91 – -1.05) |  |
| Disagree/strongly disagree | -8.47 (-11.63 – -5.32) | <0.001 |
| **I am using other resources for learning from home** |  |  |
| Strongly agree | Ref |  |
| Agree | -9.89 (-4.46 – 2.48) |  |
| Neutral | 0.49 (-3.61 – 4.58) |  |
| Disagree/strongly disagree | -0.02 (-3.64 – 3.68) | 0.72 |
| **My child has access to their own laptop/computer/tablet/smartphone to complete school work** |  |  |
| Strongly agree | Ref |  |
| Agree | -3.47 (-5.55 – -1.38) |  |
| Neutral | -4.34 (-8.97 – 0.29) |  |
| Disagree/strongly disagree | -8.82 (-12.95 – -4.68) | <0.001 |
| **My child has access to their own desk and workspace for school work** |  |  |
| Strongly agree | Ref |  |
| Agree | -3.17 (-5.51 – -0.83) |  |
| Neutral | 0.22 (-3.60 – 4.04) |  |
| Disagree/strongly disagree | -4.50 (-7.63 – -1.37) | <0.01 |
| **I am also working from home at the same time** |  |  |
| Strongly agree | Ref |  |
| Agree | -3.17 (-5.76 – -0.58) |  |
| Neutral | 1.88 (-2.12 – 5.87) |  |
| Disagree/Strongly agree | 1.42 (1.64 – 4.49) | 0.02 |
| **Working from home at the same time as supporting my child with learning is easy to coordinate** |  |  |
| Strongly agree/agree | Ref |  |
| Neutral | -4.35 (-7.66 – -1.04) |  |
| Disagree | -6.26 (-9-8.97 – -3.56) |  |
| Strongly disagree | -11.16 (-13.93 – -8.39) | <0.001 |
| **I am having regular contact with my child’s school and/or teacher** |  |  |
| Strongly agree | Ref |  |
| Agree | -7.47 (-11.11 – -3.83) |  |
| Neutral | -6.65 (-10.57 – -2.73) |  |
| Disagree/strongly disagree | -8.81 (-12.41 – -5.20) | <0.001 |
| **I feel supported by my child’s school and/or teacher and know who to contact if I have any problems with learning from home** |  |  |
| Strongly agree | Ref |  |
| Agree | -7.44 (-10.26 – -4.62) |  |
| Neutral | -7.91 (-11.14 – -4.69) |  |
| Disagree/strongly disagree | -10.63 (-13.75 – -7.51) | <0.001 |
| **My child’s school and/or teacher has set deadlines for my child’s work** |  |  |
| Strongly agree | Ref |  |
| Agree | -4.72 (-7.28 – -2.15) |  |
| Neutral | -2.15 (-5.60 – 1.30) |  |
| Disagree/strongly disagree | -4.66 (-7.69 – -1.63) | 0.001 |
| **I feel capable and confident in supporting my child with learning from home** |  |  |
| Strongly agree | Ref |  |
| Agree | -6.86 (-9.79 – -3.92) |  |
| Neutral | -8.95 (-12.21 – -5.69) |  |
| Disagree/strongly agree | -12.25 (-15.31 – -9.19) | <0.001 |
| **I feel capable and confident using technology to support my child with learning from home** |  |  |
| Strongly agree | Ref |  |
| Agree | -4.39 (-7.03 – -1.76) |  |
| Neutral | -5.10 (-8.31 – -1.88) |  |
| Disagree/strongly disagree | -8.94 (-12.40 – - 5.48) | <0.001 |
| **My child is engaged with and enjoying learning from home** |  |  |
| Strongly agree | Ref |  |
| Agree | -3.65 (-6.67 – -0.63) |  |
| Neutral | -4.30 (-7.52 – -1.09) |  |
| Disagree/strongly disagree | -9.25 (-12.30 – -6.20) | <0.001 |
| **My relationship with my child has improved whilst learning from home** |  |  |
| Strongly agree | Ref |  |
| Agree | -5.51 (-9.15 – -1.88) |  |
| Neutral | -7.59 (-11.19 – -3.99) |  |
| Disagree/strongly agree | -12.31 (-16.23 – -8.39) | <0.001 |
| **My child’s mental health and wellbeing has been affected by learning from home** |  |  |
| No | Ref |  |
| Yes | -6.92 (-8.78 – -5.05) | <0.001 |
